# A Multimodal Micro-Optrode Combining Field and Single Unit Recording, Multispectral Detection and Photolabeling Capabilities

**DOI:** 10.1371/journal.pone.0057703

**Published:** 2013-02-28

**Authors:** Suzie Dufour, Guillaume Lavertu, Sophie Dufour-Beauséjour, Alexandre Juneau-Fecteau, Nicole Calakos, Martin Deschênes, Réal Vallée, Yves De Koninck

**Affiliations:** 1 Unité de neurosciences cellulaires et moléculaires, Institut universitaire en santé mentale de Québec, Québec, Québec, Canada; 2 Centre d’optique, photonique et laser, Université Laval, Québec, Québec, Canada; 3 Department of Psychiatry & Neuroscience, Université Laval, Québec, Québec, Canada; 4 Center for Translational Neuroscience, Duke University Medical Center, Durham, North Carolina, United States of America; Institute for Interdisciplinary Neuroscience, France

## Abstract

Microelectrodes have been very instrumental and minimally invasive for *in vivo* functional studies from deep brain structures. However they are limited in the amount of information they provide. Here, we describe a, aluminum-coated, fibre optic-based glass microprobe with multiple electrical and optical detection capabilities while retaining tip dimensions that enable single cell measurements (diameter ≤10 µm). The probe enables optical separation from individual cells in transgenic mice expressing multiple fluorescent proteins in distinct populations of neurons within the same deep brain nucleus. It also enables color conversion of photoswitchable fluorescent proteins, which can be used for post-hoc identification of the recorded cells. While metal coating did not significantly improve the optical separation capabilities of the microprobe, the combination of metal on the outside of the probe and of a hollow core within the fiber yields a microelectrode enabling simultaneous single unit and population field potential recordings. The extended range of functionalities provided by the same microprobe thus opens several avenues for multidimensional structural and functional interrogation of single cells and their surrounding deep within the intact nervous system.

## Introduction

Microelectrode recordings are largely used *in vivo* to study cell physiology in intact brain. Unlike imaging techniques, they are not limited in depth measurements. Indeed, two-photon microscopy, for example, has been very instrumental to exploit the capabilities opened by fluorescent sensors for *in vivo* imaging, but the approach remains restricted to surface measurements (<1000 µm for brain tissue) [1], limiting its applicability for *in vivo* studies from deep brain structures. Microelectrodes on the other hand, allow for very long term measurement in minimally invasive condition, but are limited in types of information they provide and do not allow distinguishing from cells that present similar electrophysiological signatures. Here we show how multiple sensing capabilities can be embedded in a single aluminum coated glass microprobe. The design presented here extends on a recently developed microprobe made of a dual core tapered optical fibre. One core is a standard optical core used to excite and collect fluorescence and the second is a hollow core filled with an electrolyte allowing extracellular single unit electrophysiological recordings [2].

Fluorescent labeling offers the advantage of having relatively narrow absorption and emission spectra and fluorescent proteins spectra now cover the entire visible optical spectrum [3], [4]. It is thus possible to combine two or more fluorescent markers within the same sample, as long as their spectra are not overlapping. Here we demonstrate that the probe can perform simultaneous multispectral optical recordings *in vivo* to detect similar cell types expressing different fluorescent proteins within the same brain nucleus. We also show that using photoconvertible fluorescent proteins [5], the microprobe can be used to convert the fluorescence properties of a cell to photolabel it. This work also demonstrates that a thin reflective aluminum film can be used as a large scale field potential electrode yielding a highly compact microprobe with combined single unit and population recording capabilities. This allows relating activity in single cells to that of the surrounding population of neurons. These capabilities significantly enhance the amount of information that can be probed with minimal disruption of tissue for *in vivo* studies and no limit in depth at which measurements can be made.

## Materials and Methods

### Ethics Statement

All protocols were performed in accordance with the guidelines from the Canadian Council on Animal Care and were approved by the Laval university animal ethics committee (approval ID 2011008-1). Throughout the housing period, animals were housed in the animal facilities of the Institut universitaire en santé mentale de Québec in pairs (for rats) our in groups of 2 to 5 (for mice) to ensure social enrichment. Material enrichments like nesting material, plastic tube or dome were also provided. To prevent pain and discomfort animals were maintained in surgical plane of anesthesia during all surgical procedures and recording sessions as described in the animal preparation section. After recording sessions, animals were sacrificed with a lethal ketamine-Xylazine dose.

### Microprobe and Optical Setup

**Figure 1:**
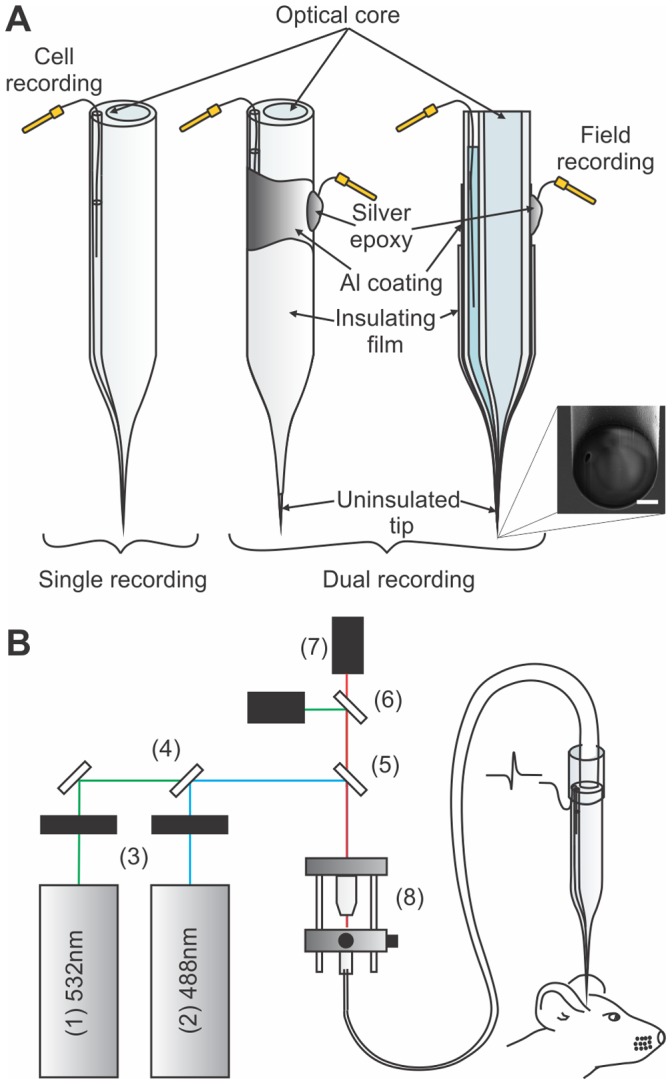
Optical and electrical microprobes. a) Schematic representation of the probe (left) and a metal coated probe adapted to achieve large field recording through the Al coating (middle: 3D representation, right: transverse cut view). Insets are scanning electron microscopy images of the respective electrode tips (scale bars are 2 µm). b) Experimental setup for multispectral detection showing : (1) 543 nm laser (25-LGR-193-249, Melles Griot), (2) 488 nm laser (FCD488 24 mW, JDS Uniphase Corporation), (3) shutters (LS3,Uniblitz), (4) 495 nm dichroic mirror (495DCLP, Chroma Technology Corporation), (5) multiline dichroic mirror (51015bs, Chroma Technology Corporation), (6) 495 nm dichroic mirror (495DCLP, Chroma Technology Corporation), (7) PMT detectors (H6780-20, Hamamatsu) and bandpass filters (ET520/40M and ET005/52M, Chroma Technology Corporation) and (8) objective (UIS-2 Plan-N, NA = 0.25, Olympus Corporation) and the fibre optic launch system (KT110, Thorlabs Inc.).

Probes were made from tapered dual core optical fibre designed in collaboration with the INO (Québec, Canada, http://www.ino.ca). Schematics of the probes are shown in **Fig. 1a**. and fabrication details were given elsewhere [2]. Briefly, the probe is made from a tapered multimode fibre that combines an optical core for optical illumination and detection as well as a hollow core serving as an electrode Probes tips were cleaved to a diameter of 10 microns, were filled with 2 M NaCl and connected to the optical setup via a relay fibre (**Fig. 1b**). In some experiments, an aluminum (Al) coating (100 nm) was evaporated on the probe shoulder and shank using a vacuum metal evaporation system. Aluminum was chosen to act both as an optical reflector and an electrical conductor to prevent optical losses through the tapered region and allow electrical recording. Its resistance to oxidation yielded more stable optical and electrical properties. A 100 µm diameter stainless steel wire was attached to the coating with silver epoxy (H2OE, Epoxy Technology). Conductive metallic films were isolated with UV curing adhesive (NOA81, Norland products). Only 100–250 µm at the tip of the probe remained uncovered to serve as an electrode. A dual wavelength detection system was used to differentiate labeled cell types on the basis of their fluorescence spectrum. The optical setup used with the probe and its components is described in **Fig. 1b**. Photomultiplier tube (PMT) detector outputs were amplified (Gain = 0.5 to 10), filtered (10 Hz low pass, Model 440, Brownlee Precision Co.) and stored on disk.

### Animals

Experiments were conducted on adult male Sprague-Dawley rats (n = 4 rats) (250–350 g) anesthetized with ketamine (75 mg/kg)-xylazine (5 mg/kg) or on mice (n = 2 mice) anesthetized with ketamine (100 mg/kg)-xylazine (10 mg/kg). Animals were placed into a stereotaxic apparatus. The animal breathed freely and body temperature was maintained at 37.5°C with a heating pad controlled thermostatically. During the experiment, the animal was maintained under deep anesthesia using additional doses of anesthetics given at 1 h interval. A craniotomy was performed over the region of interest where the probe was lowered using a micromanipulator (Burleigh 8200, Inchworm).

### Optical and Electrical Recordings

The hollow core of the optrode was used to perform single unit recordings and in a subset of experiments metal coatings were used to record the activity of a large volume of surrounding cells. Field potential and single unit recordings were amplified (Neurodata IR183, Cygnus Technology) and filtered (bandpass 0.2–300 and 200–3000 Hz respectively, Model 440, Brownlee Precision Co.) For multicolour detection experiments (**Fig. 1b**), two different excitation sources were needed (a HeNe, 5 mW, 543 nm from Coherent and a FCD488, 24 mW, 488 nm from JDS Uniphase Corporation; **Fig. 1b** items 1–2). A shutter (LS3, Uniblitz) was placed in front of each source to continuously alternate (at a ≅ 2 Hz frequency) between the two excitation wavelengths (**Fig. 1b**, item 3). The first laser was reflected on a mirror and passed through the dichroic mirror (495DCLP, Chroma Technology Corporation; **Fig. 1b**, item 4) before being reflected by a multiline dichroic mirror (51015bs, Chroma Technology Corporation; **Fig. 1b**, item 5). The second laser reflected on the first dichroic mirror and on the second. Fluorescence was collected and transmitted by the multiline dichroic mirror. The fluorescence was then reflected (for the green fluorescence) or transmitted (for the red fluorescence) by a third dichroic mirror (495DCLP, Chroma Technology Corporation; **Fig. 1b**, item 6) toward two different PMT detectors (**Fig. 1b**, item 7). Again, two bandpass filters were placed in front of each detector to isolate the sampled fluorescence (ET520/40M and ET005/52M, Chroma Technology Corporation). Fluorescence and the electrophysiological recordings were simultaneously digitized and stored on disk. PMT detector outputs were amplified (Gain = 0.5 to 10) and filtered (10 Hz low pass, Model 440, Brownlee Precision Co.) as mentioned in the optical setup section.

### Cell Cultures

To demonstrate multicolour detection, *in vitro* experiments were conducted on hippocampal cultured neurons (14 days old) previously transfected with tdTomato or mGFP. 24 hours prior to the experiments, cells were placed in a well containing 0.5 ml of neurobasal (NB, 21103-049) medium supplemented with B-27 (1∶50, 17504-044), Penicillin-Streptomycin (1∶200, 15140-122) and Glutamax (1∶400, 35050-061) and 100 µL of NB containing 0.5 µg of DNA and 2 µL of lipofectamine (11668) was deposited in the well for five hours incubation, after what cells were incubated in supplemented NB until experimentation. All culture medium chemical were purchased from Invitrogen Inc. Cells were fixed in 4% paraformaldehyde for 10–20 min and placed in a microscope recording chamber filled with phosphate buffer (0.1 M). The optical probe was mounted on a manipulator (ROE-200, Sutter Instrument Company) and moved towards fluorescent cells under visual guidance using an Eclipse E600FN, Nikon microscope. The fluorescence signals of the two different PMTs were recorded simultaneously as the probe was moved in front of the cells.

### Multicolour Transgenic Mice

*In vivo* experiments were conducted on BAC transgenic mice where neurons expressing tdTomato and EGFP proteins under the control of the promoters for D1 and D2 dopamine receptors, respectively (*Drd1a*-tdTomato/*Drd2*-EGFP transgenic mice [6]–[8], courtesy of Nicole Calakos and Jean-Martin Beaulieu). A craniotomy was performed over the frontal part of the striatum (where dopaminergic inputs are abundant). Probes were lowered into the striatum to depths ranging from 1500–2500 µm while red and green fluorescence were simultaneously recorded along with the electrophysiological signal.

### Photoconversion

*In vitro* experiments were also conducted in hippocampal mEOS2 transfected cells. Protocol for cultured cell handling and transfection was similar to the one presented in previous section. But in this case, cells were not fixed and were kept alive under microscope with the help of a constant ACSF perfusion (120 mM NaCl, 0.6 mM CaCl2, 5 mM MgCl2, 2.5 mM KCl, 10 mM Hepes (7.9) and 8 mM D-Glucose). For these experiments, a conventional mercury lamp (Wild Leitz Canada) was filtered (Thorlabs, FP400-40), collimated and injected through the microprobe. Emission properties of mEOS2 were converted using UV illumination provided by the microprobe. Fluorescent red and green signals were image separately (Zeiss filter sets: (BP564/12, FT580, LP590) and (BP450-490, FT510, BP515-565) under a microscope objective to visualize in real time the emission spectral shift of the mEOS2 as it was being exposed to UV light through the microprobe. Red fluorescence was continuously recorded, and to insure that the increase was due to conversion, green fluorescence was measured at two different time points during photo-conversion.

### Quantification of Light Collection through the Microprobe Wall

The Al coating serves as electrodes but they also prevent light losses through the tapered wall of the probe. To quantify the side collection of uncoated probe, the tapered region of an Al-coated probe was soaked into a (10 mM) Lucifer Yellow 4% Agar solution and the fluorescence collected was recorded. The metal coating was then chemically attacked using a basic solution (NaOH, 0.1 M). Finally, the probe was soaked again into the same fluorescent solution and the collected signal was re-evaluated. This technique allowed us to compare the exact same probe without having to change the conditions for light injection in to the probe.

### Data Analysis

To show the correlation between single unit and local field potential activity, spikes were computed in time histograms where the time point *t* = 0 corresponds to the maxima of the slow component of the local field potential (2–10 Hz). Spikes were computed for 15 field cycles.

Fluorescence values are commonly presented as the variation of fluorescence divided by the initial fluorescence level (ΔF/F_0_). In a vertical scanning protocol, as the one we use here, the value of F_0_ can be very low or vary as a function of the probe penetration in tissue and we opted to present the fluorescence data as the variation of fluorescence intensity divided by the peak-to-peak root mean square (RMS) value of the initial fluorescence (signal-to-noise (S/N)). Details on the cell detection protocol and criteria were described elsewhere [2].

## Results

### Multispectral Detection

**Figure 2:**
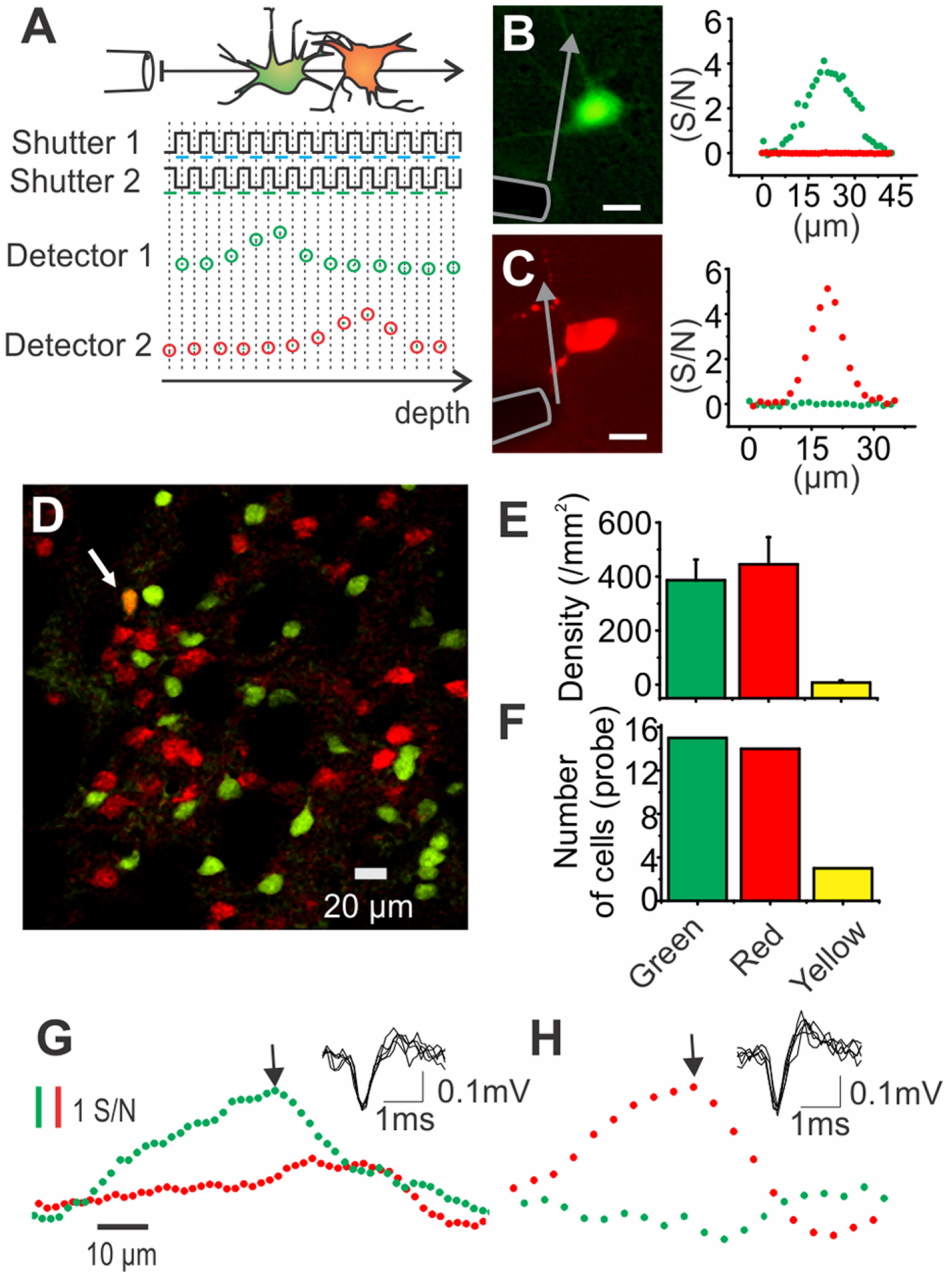
Multispectral detection allows identifying D1 and D2 expressing neurons. A) Schema of the shutters state and incident signals on the detecting PMTs as the probe passes by a green and a red cell in succession. B) (Left) Micrograph of a GFP fluorescent cell and a microprobe (highlighted with gray contour). Scale bar is 10 µm. (Right) Collected fluorescence in the PMT detector 1 (green dots), and 2 (red dots) as the probe is moved transversally in front of the cell shown in left panel. C) Same result is shown as the probe pass by a tdTomato fluorescent cell. Scale bar is 10 µm. Arrows in (B) and (C) represent the probe displacement in front of the cell. D) Striatal section of a transgenic mouse showing cells expressing fluorescent proteins under the control of D1 (red) or D2 (green) receptor (green) promoters. Some cells co-express both fluorescent proteins (see arrow). E) Average density of the different cell types in the striatum (n = 5 striatal sections). Cells were counted and densities were estimated with the help of the software Image J. F) Histogram of the optically detected cells. Fluorescent cell detections were accompanied by a rise (signal-to-noise >2) and a decay of green and/or red fluorescence as described previously. A total of 29 cells were detected and computed in a histogram according to their fluorescence colour. (G-H) Examples of *in vivo* simultaneous optical and electrical recordings (inset) as the probe pass by a green (G) or a red (H) cell.

Multispectral detection is used in microscopy to discriminate cells labeled with proteins having different emission spectra. To test the compatibility of multispectral detection with our microprobe, we injected light from two different excitation sources in the probe optical channel. The detection system proposed here (see schematics in **Fig. 2a**) was successfully used to differentiate two cell populations labeled with two different fluorescent markers *in vitro* and *in vivo*. Recorded fluorescence signals as a function of the probe position in front of a fixed GFP cell is shown in **Figure 2b**. The same result is shown for a tdTomato cell (**Fig. 2c**). Fluorescence increase and decrease only in the corresponding PMT signal as the probe passed the cell. To test the ability of the probe to differentiate between two labeled populations of cells *in vivo*, we performed vertical scanning experiments through the striatum of adult transgenic mice expressing tdTomato and EGFP proteins under the control of the D1 and D2 dopamine receptor promoters, respectively [6]–[8]. Probes descents were repeated within the following setreotaxic coordinates: 1.5 to 2.5 mm lateromedial, 0.5 to 1.5 anterior to Bregma and 2 to 3.5 mm below brain surface. **Figure 2d and 2e** shows the distribution of tdTomato and EGFP in the striatum. Cells expressing the D1 and D2 receptors could be clearly identified (**Fig. 2d**) and very few cells expressed both markers. Identification of D1 and D2 cells with the microprobe yielded a similar distribution of D1-only, D2-only and combined D1/D2 cell detection as that obtained under microscopy analysis (**Fig. 2f**). Optical detection of EGFP and tdTomato expressing cells respectively is shown in **Figure 2g–h** along with five superimposed consecutive spikes recorded at fluorescence peaks. The microprobe thus provides a means to identify and record from different labeled populations of cells intermingled within the same deep brain structure.

### Photoconversion

**Figure 3:**
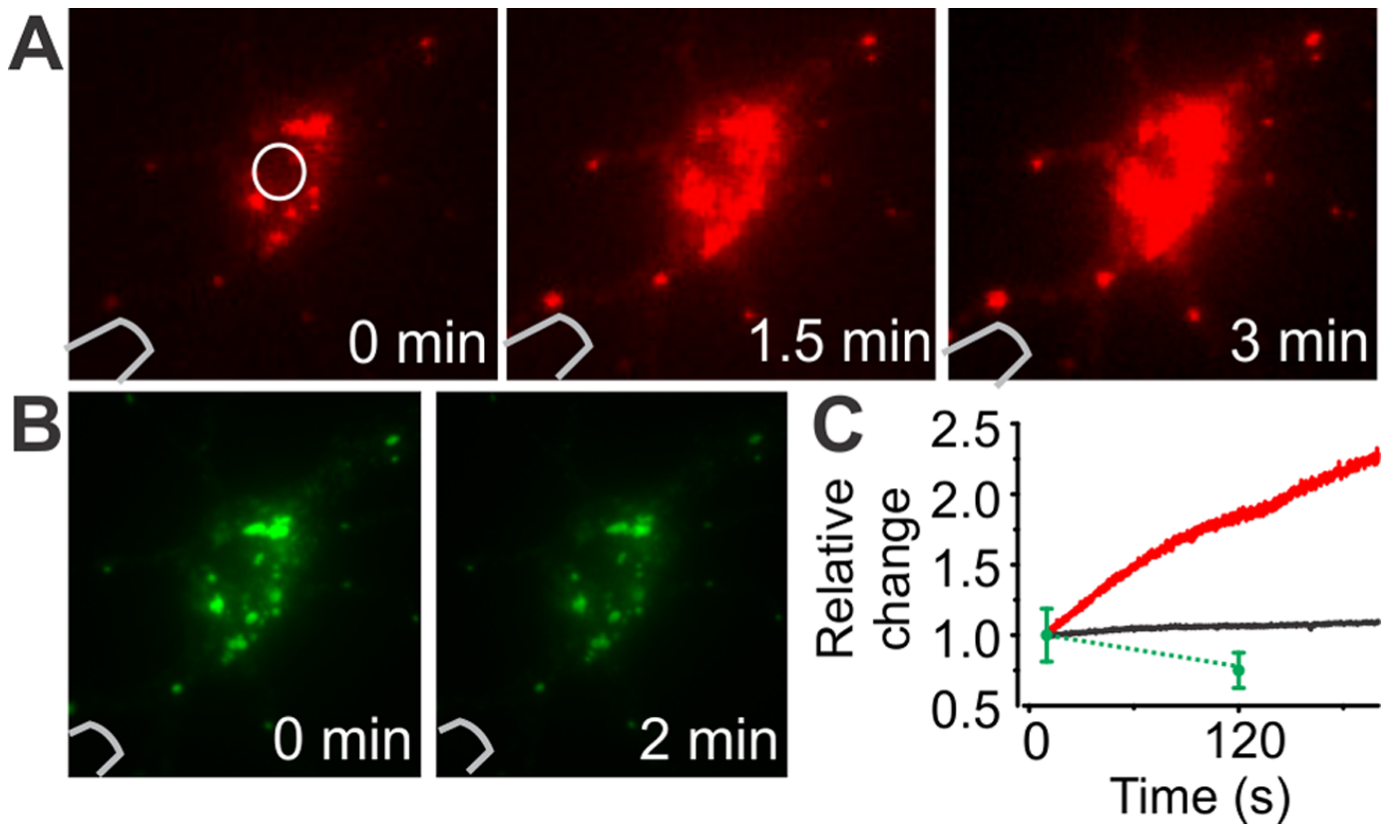
Photoconversion of mEOS. A–B) Images at different time points of a mEOS2 expressing cell during UV-induced photoconversion. UV illumination with the probe causes an increase in red fluorescence (A) and a decrease in green signal (B). C) Red and green emission of a cell during photo-conversion as a function of time. The region of interest taken into account is shown in (A) (white circle). Note that only two time points were measured for the green signal. Change in background fluorescence around the cell is shown in black.

To test whether the microprobe could be used to change emission properties of photo-switchable fluorescent proteins, we illuminated mEOS2 transected cells with UV light injected through the microprobe. Intensity at probe tip used to convert emission properties was estimated to 2 mW/mm^2^. Results show a significant increase in red signal and a decrease in green fluorescence of an isolated mEOS2 expressing neuron (**Fig. 3**). This indicates that the microprobe is suitable to label by photo-conversion or photo-activation *in vivo* recorded cells.

### Light Collection through the Microprobe Wall

**Figure 4:**
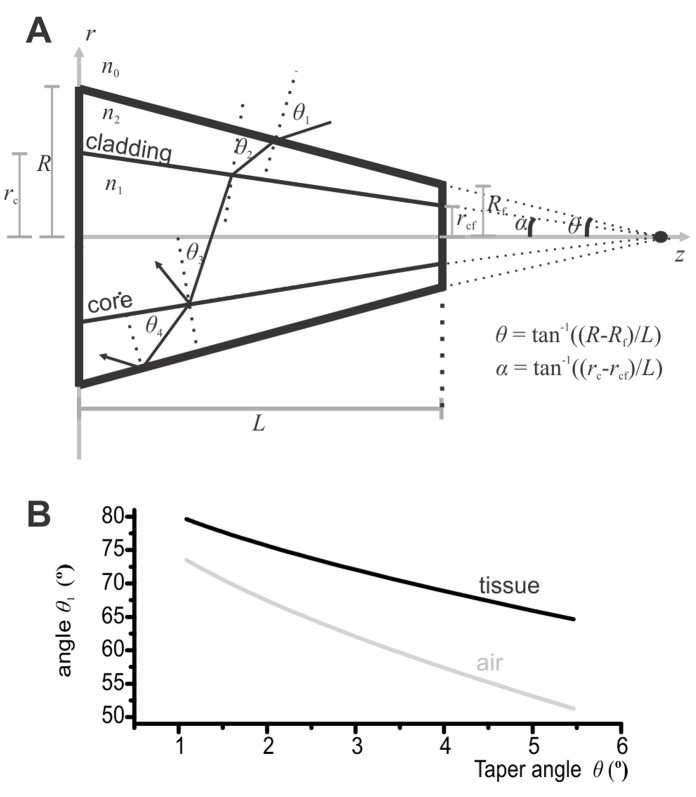
Side acceptance of tapered waveguides. A) 2D schematic representation of ray acceptance within a tapered waveguide (thick black boundaries). Note that for visualization purpose the taper angle was exaggerated in this illustration. B) Critical accepted incidence angle *θ*_1c_ as a function of the taper angle *θ*. Rays with incidence angles ranging from *θ*_1c_ to 90° will be accepted in the waveguide (parameters were fixed as follows: *R* = 100 µm, *R*_f = _5 µm, *r*_c = _60 µm, *r*_cf_ = 3 µm, *n*_0_ = 1 (air), *n*_0_ = 1.35 (tissue) *n*_1_ = 1.47 and *n*_2_ = 1.45).


Tapered waveguides can collect light through their wall with an acceptance angle that varies with the taper angle *θ*. Wall collection is less effective per surface area than tip collection, but the surface of the tapered region is significantly larger than the tip transversal surface. **Figure 4** illustrates light behavior as a ray is incident on the wall of a tapered waveguide. *θ* and *α* are the half angles of the cladding and core tapers. They are determined with the taper length *L* as well as the initial (*R* and *r*_c_) and final (*R*_f_ and *r*_cf_) dimensions of the waveguide. These parameters are defined in **Figure 4**. The ray encounters four interfaces with incidence angles *θ*_1_, *θ*_2_, *θ*_3_ and *θ*_4_ respectively. The refractive index of the external medium, the waveguide core and cladding are defined as *n*_0_, *n*_1_ and *n*_2_. For small incidence angle *θ*_1_ the ray will be transmitted through the tapered waveguide, but for large *θ*_1_, the ray will undergoes total internal reflection at the interface between the waveguide core and the cladding (if *θ*_1_ is big enough) or at the interface between the cladding and the external medium. In the later case, the ray will be first guided in the cladding, and after few reflections its incidence angle in the waveguide core will fulfill the total internal reflection requirement of the core. From Snell-Descartes law we can calculate the critical angle *θ*_4c_ at the interface between the cladding and the external medium and evaluate the angle *θ*_1c_ that will lead to that critical angle.

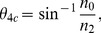
(1)


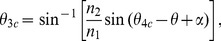
(2)


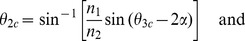
(3)


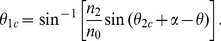
(4)

The critical accepted incidence angle *θ*_1*c*_ as a function of the taper angle *θ* was calculated for our probe parameters (*R* = 100 µm, *R*_f = _5 µm, *r*_c = _60 µm, *r*_cf_ = 3 µm, *n*_1_ = 1.47 and *n*_2_ = 1.45.) in air (*n*_0_ = 1) and in tissue (*n*_0_ = 1.35). **Figure 4b** shows that a significant range of incidence angles along the shank of the probe will enter and remain within the waveguide. Note that for angles that are not satisfying the total internal reflection condition, partial reflection will occur. We therefore made a batch of probes coated with a thin reflective layer of Al to block light collection through the probe shank (**Fig. 1a**).

### Effect of Aluminum Coating on Background Fluorescence Noise

**Figure 5:**
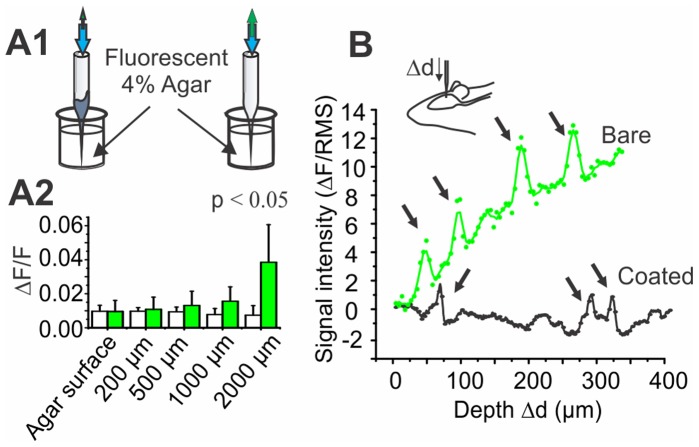
Impact of fluorescence collection through the microprobe walls. A1) Schematics of the coated and uncoated microprobe descents into fluorescent agar. A2) Fluorescence measurements for different penetration depths into fluorescent agar for bare (green) and coated (white) probes (n = 5 probes; 1°<*θ* <3°)). B) Effect of the coating on the fluorescence DC level when a bare (green) or a coated (black) probe is lowered into cortex and thalamic issue. Arrows show location of fluorescent cells (inset: representation of the probe displacement).

To ascertain the value of coating the probes with a reflective film, we measured experimentally the impact of fluorescence collection through the microprobe wall. For this, we measured fluorescence collected from coated versus uncoated probes as they were dipped into a uniform fluorescent medium. For uncoated probes, the fluorescence increased as a function of the penetration depth whereas this was not the case for the coated probes (**Fig. 5a**). Thus Al coating effectively prevents fluorescent signal contamination from the side of the microprobe. We then tested fluorescence changes as the microprobe is advanced through tissue containing individual labeled cells. Transient rise and decay in fluorescence occurred as the probe passed by labeled cells as previously described [2]. These transients were similar with the coated and uncoated probes. However, wall collection caused a gradual shift in the signal DC level with the bare probes (**Fig. 5b**). In a static protocol, where the probe position is maintained and fluorescence is used to measure functional events such as fluctuations in ionic concentration, wall collection must be considered when choosing the experimental conditions (*e.g.*, labeling volume and sparseness, probe tapering angle). The coating also helps minimize photobleaching and photodamage due to light escaping from the side of the probe. In contrast, the presence of coating does not improve individual cell detection when using the dynamic vertical scanning protocol described previously [2].

### In vivo Simultaneous Field Potential and Single Unit Recordings

**Figure 6:**
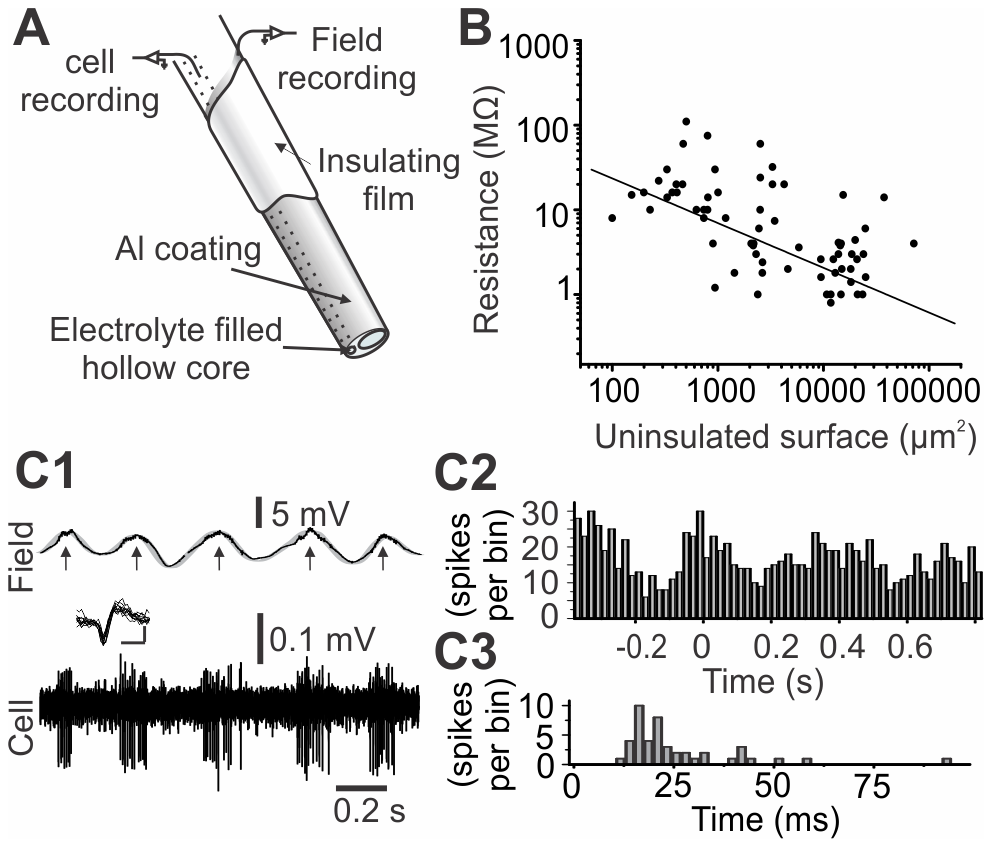
Coated glass microprobes enable dual electrical recordings. A) Schematic representation of a multimodal microprobe tip. B) Measured resistance as a function of the uninsulated surface. C) Simultaneous recording of field potential oscillations and single unit achieved with the microprobes. Spikes were computed in a time histogram (C1). Inset: overlay of 10 successive spikes (vertical scale bar: 0.1 mV, horizontal scale bar: 1 ms) (C2) according to time of occurrence relative to field maxima (arrows in c1; n = 15) and into an interspike interval histograms (C3).

Given the conductive properties of Al, coatings can also serve as field potential electrodes. The detecting surface (uninsulated tip) of these electrodes must be relevant to the size of the region of interest and their resistance must be as small as possible. To characterize probe performances, we measured electrode resistance for different detecting areas. Thin metallic layers may have very high resistances, but the thickness used here (≈ 100 nm) is large enough and has as a negligible effect on the electrode resistance. The resistance is therefore low enough for field potential recordings and can be adjusted as desired by controlling the uninsulated tip surface of the electrode and, as predicted by the Robinson model [9], is inversely proportional to that surface (**Fig. 6a**, R^2^ = 0.95, n = 74 electrodes). Microprobes with metal coating resistances ranging from 0.6 to 4 MΩ were used to record simultaneously single unit (through the hollow core) and field potentials during spontaneous activity (n = 28 cells) (**Fig. 6c**). In **Figure 6c**, field and single unit recordings show the typical slow wave activity of cortical neurons under ketamine-xylazine anesthesia [10]. Note that this frequency differs from anaesthetized rat respiratory frequency (typically 1 Hz). **Figure 6c** also shows a histogram where spikes were computed according to the slow field cycles (2–10 Hz) and an interspike interval histogram. Spike occurrence shows a correlation with the field state. This dual recording capability adds new functionality to this type of fibre optic microprobe, including the ability to interrogate the central nervous system both at single cell and network levels concurrently.

## Discussion

The present work shows how a unique micro-optrode can combine single unit and large scale field recordings. It also shows that the probe optical channel is suitable for multicolour detection.

Fluorescent proteins are now covering the majority of the visible spectrum [4] and consequently, more than one cell types can easily be labeled within the same animal with a specific genetic colour coding. Using multi-excitation sources and detectors we showed that the same probe can be used to simultaneously identify and record two different cell populations at depth inaccessible to conventional two-photon microscopy [1]. Alternative techniques are being developed to reach deeper regions of the brain such as micro-endoscopy, but they remain relatively invasive and they do not allow direct electrical single unit recordings [11], [12]. Additionally, the multispectral capabilities presented here open possibilities for ratiometric dye measurements (*e.g.*, with the calcium indicator Fura-2) or for spectral measurements of frequency resonance energy transfer (FRET) [13].

Our experiments also demonstrate that probes can be used to convert fluorescence of photoconvertible fluorescent proteins to label recorded cells *in vivo*. Labeling recorded cells can be extremely valuable for post-hoc morphological or biochemical characterization.

This work also demonstrated that reflective metal films on the microprobe while reducing fluorescent contamination in some protocols and architectures can be used to perform field potential recordings. Thus the probe can be used to simultaneously acquire single cell recordings and field potentials from large number of cells. Knowing the overall activity of the population can be valuable especially when using a stimulation protocol; it can therefore be very instrumental to compare the cell response to the population response. One advantage of the probe is that the field recording electrode is located very near the single cells recording component ensuring that both signals are centered at the same point even though they represent different sampling volumes. Coupled with the ability to identify cells based on their fluorescence this can be particularly enabling to contrast the response of sparsely distributed genetically modified cells (*e.g*, after in utero electroporation) to that of the surrounding population [14], [15].

Controlling the sampling volume can be achieved by modifying the impedance properties and recording surface of the field electrode provided by the coating. To achieve this, the impedance can be adapted using different tapering shapes and insulation length as well as by adding spongy or rough deposit (to modify surface contact [9]).

In conclusion, the combined optical and electrical multimodalities of the probe and its micrometric resolution vastly expand the possibilities for *in vivo* electrophysiology, in particular, with optical means to monitor the impact of genetic manipulations of individual cells *in vivo*. The simplicity of the probe, its low cost, ease of use and adaptability make it particularly attractive modality to enhance *in vivo* electrophysiological recording systems.
